# A Method to Assess the Potential Effects of Air Pollution Mitigation on Healthcare Costs

**DOI:** 10.1155/2012/935825

**Published:** 2012-09-11

**Authors:** Bjørn Sætterstrøm, Marie Kruse, Henrik Brønnum-Hansen, Jakob Hjort Bønløkke, Esben Meulengracht Flachs, Jan Sørensen

**Affiliations:** ^1^Centre for Applied Health Services Research and Technology Assessment, University of Southern Denmark, J.B. Winsløws Vej 9B, 1, 5000 Odense C, Denmark; ^2^The Danish Institute for Health Services Research, Dampfærgevej 27-29, 2100 Copenhagen, Denmark; ^3^Department of Public Health, University of Copenhagen, Øster Farimagsgade 5, 1014 Copenhagen, Denmark; ^4^Institute of Public Health, Aarhus University, Bartholins Allé 2, 8000 Aarhus C, Denmark; ^5^National Institute of Public Health, University of Southern Denmark, Øster Farimagsgade 5A, 2, 1353 Copenhagen, Denmark

## Abstract

*Objective*. The aim of this study was to develop a method to assess the potential effects of air pollution mitigation on healthcare costs and to apply this method to assess the potential savings related to a reduction in fine particle matter in Denmark. *Methods*. The effects of air pollution on health were used to identify “exposed” individuals (i.e., cases). Coronary heart disease, stroke, chronic obstructive pulmonary disease, and lung cancer were considered to be associated with air pollution. We used propensity score matching, two-part estimation, and Lin's method to estimate healthcare costs. Subsequently, we multiplied the number of saved cases due to mitigation with the healthcare costs to arrive to an expression for healthcare cost savings. *Results*. The potential cost saving in the healthcare system arising from a modelled reduction in air pollution was estimated at €0.1–2.6 million per 100,000 inhabitants for the four diseases. *Conclusion*. We have illustrated an application of a method to assess the potential changes in healthcare costs due to a reduction in air pollution. The method relies on a large volume of administrative data and combines a number of established methods for epidemiological analysis.

## 1. Introduction

Air pollution is known to have an adverse effect on different sectors of the economy, including public health. Air pollution causes increased morbidity and mortality [1]. It is therefore relevant to include in cost-benefit analyses of mitigation projects an assessment of the potential savings in healthcare resources due to a reduction in morbidity and mortality.

The relationship between exposure to air pollution, the disease burden, and healthcare costs cannot be determined precisely. Air pollution impacts negatively on health; mitigating air pollution could potentially result in health benefits. A recent literature review identified several studies of the relative changes in incidence and mortality associated with changes in air pollution [1]. The review specifically identified the connection between an increase in fine particulate matter (PM2.5) and the following four diseases: coronary heart disease (CHD), stroke, chronic obstructive pulmonary disease (COPD), and lung cancer [1]. A review linked to the present study that explored the literature relating to healthcare cost savings of a reduction in air pollution identified six nonsource-specific studies on the impact of particulate matter on healthcare costs [2–7]. These studies were conducted in other countries and dealt with air pollution in different circumstances. They were prevalence-based and focused on coarse particulate matter (PM10). PM10 is associated with less severe health effects than is PM2.5 [8].

In the present study, we chose to focus our analysis on the four diseases mentioned above. The objective was to develop a method to assess the potential effects of air pollution mitigation on healthcare costs. We focused on the effects of changes in the level of PM2.5 and developed a method to assess the potential benefits for the Danish healthcare sector from a reduction in air pollution. As a case, we assumed a 10 *μ*g/m^3^ decrease in the annual mean level of PM2.5. Compared with data from the Danish national air pollution monitoring programme, this corresponds to a reduction of 51% and 76% of present levels in Copenhagen at street level and rooftop urban background, respectively [9].

## 2. Materials and Methods

Since most individuals are exposed to some levels of air pollution, it is not feasible to differentiate between individuals who are exposed to certain levels of air pollution. Therefore, this study used an indirect method of identifying the “exposed” individuals (as opposed to nonexposed). By applying assumptions about the relative risks of new diseases (incidence) we attributed a change in disease incidence to a change in air pollution exposure.

In Denmark, easy access to administrative health registers provides excellent conditions for register-based research. The use of individual civil registration numbers enables researchers to make connections between registers containing information about individuals' use of healthcare resources as well as demographics and other social variables. The advantages of such registers have been well illustrated in Davidsen et al. [10]. For this study, we used individual encrypted data on healthcare utilisation, sociodemographics, education, migration, and death on the entire Danish adult population. These data were supplied and hosted by Statistics Denmark. To describe healthcare utilisation, we used data from the Danish National Health Service Register, the Danish National Patient Register, and the Danish National Prescription Registry. These registers hold detailed information about the use of healthcare resources. Information on sociodemographics, education, migration, and death was retrieved from the Danish Civil Registration System and from registers about level of education, labour market affiliation, personal income and transfer payments, and causes of death. We also used data from the DANCOS cohort [10], which includes individuals who participated in the Danish nationally representative health interview surveys. The cohort provided self-reported information on health status and smoking habits, among other things. To simplify the analyses, we excluded individuals who emigrated from Denmark after the baseline year.

Healthcare costs include utilisation of primary and secondary healthcare services and of prescription medicine. Healthcare costs in the primary healthcare sector were defined as the reimbursement to the patient by public health insurance (in most cases 100% of average costs). The diagnosis-related groups (DRG) tariff, defined as the average cost per admission, was used as the cost of admissions. The ambulatory grouping system (DAGS) tariffs defining the average cost per visit were used to cost outpatient or emergency room visits in the secondary healthcare sector. Neither the DRG nor DAGS tariffs included contributions to fixed costs. To value the cost of prescription medicine, the full sales price was used, including both the patient's payment and the reimbursed element. The currency was translated into euros using a 7.45 DKK/EUR exchange rate. Costs were discounted at a 3% discount rate and adjusted to similar pricing levels (fixed at 2006 pricing levels).

### 2.1. Assumptions of Relative Risk

For women aged 50–79 and exposed to a 10 *μ*g/m^3^ increase in the annual mean level of PM2.5, the relative risk of being incident with CHD and stroke was assumed at 1.21 and 1.35, respectively [11]. No similar data were available for the long-term effects in males. Based on findings on mortality related to particulate emissions, for example, Pope et al. [8], we assumed that the relative risk for cardiovascular events in men was lower than for women. Tentatively, therefore, we assumed the relative risk (RR) to be half that of the increased risk for women, that is, for stroke the RR for men was 1.175 and for coronary heart disease 1.105.

For both men and women aged over 30, we assumed that the RR of COPD for a 10 *μ*g/m^3^ increase in the annual mean level of PM2.5 was 1.14 [12]. For lung cancer, we used the RR of mortality as an approximation for incidence. The RR of lung cancer mortality per 10 *μ*g/m^3^ increase in the annual mean level of PM2.5 was assumed at 1.14 [13]. We assumed that, by inverting the RR, we would achieve an expression for the reduction in incidence due to a 10 *μ*g/m^3^ decline in the annual mean level of PM2.5 for CHD, stroke, COPD, and lung cancer, enabling us to calculate a number of saved cases.

### 2.2. Attributable Cost

We applied the attributable cost approach [14]. In this approach, the costs associated with a particular disease can be determined as the difference between average healthcare costs of diseased individuals and a comparable nondiseased control group, thus covering all contacts with the healthcare system regardless of the reason for the contact. In the analysis, we matched the cases (individuals with one of the four diseases) with a set of controls (individuals without any of the four diseases) by use of propensity scoring. This method aims to identify individuals who are similar with respect to a predetermined set of sociodemographic characteristics. We assumed that the cost difference for these two groups accounts for the average attributable cost of the disease.

We multiplied the average attributable cost with the number of saved cases following a reduction in air pollution to arrive at an expression for healthcare cost savings from a 10 *μ*g/m^3^ decline in the annual mean level of PM2.5.

### 2.3. Identification of Cases

Individuals were identified as cases if they had at least one inpatient admission, outpatient visit, or emergency room visit with a relevant primary diagnosis. We defined our diagnoses according to the ICD10 classification codes [15]: CHD = I20–25, stroke = I60–69, COPD = J41–44, and lung cancer = C33-34. The time of incidence was defined as the date of the first hospital contact due to disease (including death). Individuals with the disease who were in contact with primary care facilities only could not be identified. We applied a washout period of 20 years to ensure that the identification of cases was based on the first occurrence of the diagnosis.

1997 was considered as the baseline year as it was the first year where detailed data on healthcare cost was available in the registers. We required a minimum number of 25 cases for the estimates to be considered to have sufficient statistical power. Where our requirement was not met, we extended the baseline to a longer period.

According to Vestbo et al. [16], hospitalisation for COPD patients most often occurs at a relatively late stage of the disease. Therefore, we were not able to identify the true date of onset of COPD from registers. As a pragmatic solution to this, we introduced a lag-time of nine years and assumed that true incidence of COPD was nine years prior to the first hospitalisation. In this way, we identified a cohort of cases with their first hospitalisation in 2006 and followed them retrospectively from 1997 to 2005. We defined this cohort as the prehospitalisation cohort and used it in the estimation of attributable healthcare costs related to the years prior to the first hospitalisation. Regarding healthcare utilisation after hospitalisation, we used the same method as for the other three analysed diseases.

We stratified all analyses by gender and age in five-year intervals (50–54,…, 75–79, 80+). For CHD and stroke the RR change associated with an increase in the air pollution could only be identified for individuals in the 50–79 age range. We assumed that the RR for older age groups was identical to that of younger age groups. For lung cancer we excluded individuals older than 79 because the mortality by the fifth year would have resulted in fewer than 25 cases.

### 2.4. Time Horizon

The attributable costs of chronic diseases range in time from the onset of disease to the time of death. Therefore, we applied the longest time horizon allowed by the data. For CHD and stroke, the follow-up period was 10 years, for COPD 19 years, and for lung cancer eight years.

### 2.5. Matching

We matched cases with controls using the propensity scoring method [17]. In the baseline year, we determined the propensity score for the entire population at risk using logistic regression. The propensity score expresses the likelihood of being incident during the year depending on cohabiting status, educational level, socioeconomic status, age, gender, and comorbidity. For comorbidity, we used an adjusted Charlson index [18], which did not include scores achieved from the analysed diseases. We included all theoretically relevant variables that were accessible from registers regardless of their significance in the logistic regression.

**Equation 1**

Propensity score:

(1) $$\begin{array}{c} \text{logit}\left(p\right)=\ln\left(\frac{p}{1-p}\right)=\alpha_{0}+\rho X. \end{array}$$

The variable *X* in (1) included age, cohabitation status, education, socioeconomic status, and comorbidity. We excluded controls that contracted one of the analysed diseases during followup. This meant that patients were deleted from the control population. Cases were matched 1 : 5 with controls using nearest neighbour matching, where each case was matched with the five individuals closest to the case, measured on propensity score. This allowed us to identify a cohort of controls that was similar to cases (measured by the likelihood of being incident) but without having the disease.

### 2.6. Healthcare Costs

Since we had detailed data on all individuals in all years, we could apply the method developed by Lin et al. [19], dividing the entire study period into smaller periods and multiplying the Kaplan-Meier estimator for the probability of surviving to the start of each interval with an appropriate estimator of the average cost over the interval, given survival to the start of the interval. From this point on, we therefore separated regression of cases from that of controls.

Two challenges in the analysis of healthcare costs related to the distribution of costs. Firstly, some individuals had zero demand for healthcare. Secondly, given positive demand, healthcare expenditure followed a lognormal distribution. A two-part model was suitable for solving the first challenge [20]. In the first part of the two-part method we calculated the share of individuals with positive healthcare costs, *C*_*t*_^+^. We use *C*_*t*_^+^ for adjustment of the healthcare costs, which are calculated for individuals with positive healthcare costs, thus achieving an expression of the average healthcare costs for all individuals.

Given positive healthcare demand, we suggested the average cost for individuals alive at the beginning of the year to be estimated assuming a log-link and a lognormal distribution in a generalised linear mixed model with a categorical variable, *Year*, indicating the year (after incidence), and an individual random effects term, *Z*, as explanatory variables.

**Equation 2**

Two part—part II:

(2) E(Healthcare  costs ∣ positive  demand)     =α+θt∗Year+ϑ∗Z, where  θt=(θ0θ1⋮θT),

where *T* is the time horizon. With the mixed model, we addressed heterogeneity and with the generalised form we addressed the log-normality of healthcare costs. We were particularly interested in the parameter estimates of *α* and *θ*, which in combination express the log of healthcare costs.

However, since the log of healthcare costs was of little informative value, a retransformation was needed. In order to compensate for retransformation bias, we multiplied by the smearing estimator [21].

**Equation 3**

Duan smearing estimator:

(3) $$\begin{array}{c} \text{SM}=\;\frac{1}{N}\sum_{}^{}{\text{e}}^{e}, \end{array}$$

where *e* is error term from the regression of healthcare costs and *N* is the number of individuals in the cohort.

Once we knew (from (2) and (3)) the average healthcare cost given positive demand, the two-part method led to a multiplication of these findings with the share of individuals with positive demand, *C*_*t*_^+^, in order to achieve the expected healthcare cost. We did this for individuals surviving to the beginning of each year.

**Equation 4**

Expected healthcare cost for individuals surviving to the start of year *t*:

(4) $$\begin{array}{c} \text{HC}\left(t\right)=C_{t}^{+}*{\text{e}}^{\alpha_{2}+\theta_{t}}*\text{SM}. \end{array}$$

Subsequently, we calculated the survival at the end of each time period (or, equivalently, the beginning of the following time period).

**Equation 5**

Kaplan-Meier survival estimate:

(5) $$\begin{array}{c} \hat{S}\left(t\right)=\prod_{i=0}^{t}\frac{n_{i}-d_{i}}{n_{i}}, \end{array}$$

where *n*_*i*_ is number of survivors up to time period *i*, and *d*_*i*_ is the number of deaths in time period *i*.

By weighing average costs for individuals surviving to the start of period *t* with the survival to the start of period *t* (or, equivalently, end of period *t* − 1) and subsequently summing over the entire time horizon, *T*, we achieved an expression of the attributable costs of the given disease.

**Equation 6**

NPV healthcare costs per case attributable to the air-pollution-related disease:

(6) $$\begin{array}{c} \text{NPV}=\sum_{t=0}^{T}\frac{{\text{HC}}_{p}\left(t\right)*{\hat{S}}_{p}\left(t-1\right)-{{\text{HC}}_{c}\left(t\right)*\hat{S}}_{c}\left(t-1\right)}{{\left(1+\delta \right)}^{t}}. \end{array}$$

Subscripts *p* and *c* denote group assignment (patient or control),*δ* is the discounting factor and

(7) $$\begin{array}{c} {\hat{S}}_{p,c}\left(t-1\right)=1,\;\text{for}\;\;t=0. \end{array}$$

The expression in the numerator can be interpreted as the expected healthcare cost attributable to the pollution related disease in year *t*. All terms were aggregated over time in order to achieve the total net present value of expected healthcare cost attributable to the disease.

As noted previously, we assumed that the RR could be inverted to give an incidence after a 10 *μ*g/m^3^ decline in the annual mean level of PM2.5. Thus, we could calculate the decline in the incidence.

**Equation 7**

Number of Saved Cases due to a 10 *μ*g/m^3^ Decline in the Annual Mean Level of PM2.5:

(8) $$\begin{array}{c} \text{Cases}\;\;\text{saved}=\left(1-\frac{1}{\text{RR}}\right)*\text{incidence}\;\;\text{before}. \end{array}$$

Having achieved the total net present value in (8) and the number of saved cases in (9), we multiplied these two in order to achieve the benefit from mitigation in terms of healthcare cost savings.

**Equation 8**

Healthcare Cost Savings per 100,000 Inhabitants Related to a 10 *μ*g/m^3^ decline in Annual Mean PM2.5:

(9) $$\begin{array}{c} \text{Healthcare}\;\;\text{cost}\;\;\text{savings}=\text{NPV}*\text{cases}\;\;\text{saved}. \end{array}$$

## 3. Results

Table 1 shows the characteristics of the analysis populations including *t*-tests for equality of means. In the 1997 national patient register, we identified 20,083, 13,632, and 9,058 incident cases of CHD, stroke, and COPD, respectively. In 1997–1999, we identified 10,200 incident cases of lung cancer and in 2006 we identified 7,712 incident cases of COPD.

The average age spanned from 67 to 74. Of the populations, 42% to 53% were female, and this was highest for stroke and COPD, and 49% to 61% of the populations were married. The average Charlson score was lowest for the cardiopulmonary diseases, ranging from 0.3 to 0.4, while the average Charlson score for lung cancer cases and controls was about 1.2. More than 60% were unskilled and less than 10% had a university degree. More than 80% of cases and controls were outside the labour market (i.e., either retired or on early retirement). For those still in the labour market, the majority were blue collar workers.

**Figure 1:**
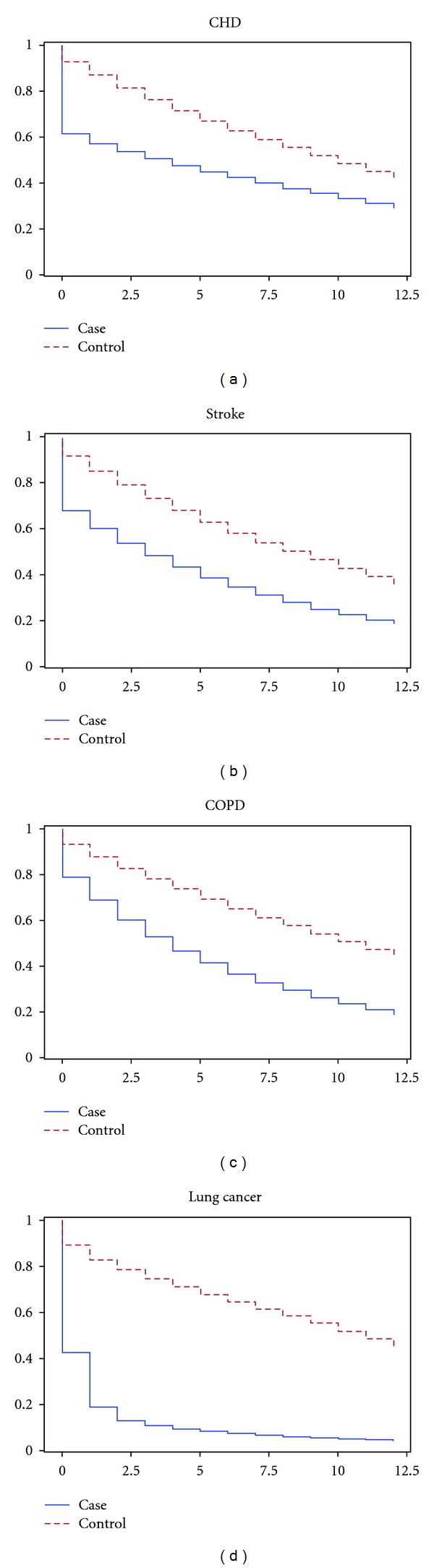
Survival curves.

Figure 1 shows the survival of the analysed populations. Not surprisingly, survival of controls was better in all four diseases. We were not able to analyse survival for COPD prior to hospitalisation. Therefore, the survival in the retrospective part of the COPD analysis was assumed at 100%.

Mortality was high among lung cancer cases. Hence, we chose to expand the baseline for lung cancer in order to increase the size of the cohort. Thus, we pooled incident cases and their controls in the years 1997–1999 for analyses of lung cancer.

Table 2 illustrates incidences per 100,000 inhabitants and attributable healthcare costs per episode. The incidences of CHD and stroke were based on 1997 data. The incidence of lung cancer was an average of incidences in 1997–1999 and the incidence of COPD was an average of 1997 and 2006. The healthcare costs per case correspond to (6).

As it is not within the scope of this study to provide a detailed analysis of the above table, it suffices to state that the incidence rates are within the expected range and in accordance with common knowledge of the epidemics of the diseases. This is also the case for the average healthcare costs. The negative attributable costs in the oldest age groups with CHD and lung cancer can be explained by a poor survival prognosis compared to their controls.

Table 3 shows the healthcare cost savings per 100,000 inhabitants of a 10 *μ*g/m^3^ decline in the annual mean level of PM2.5 for 10, 10, 19, and 8 year-time horizons for CHD, stroke, COPD, and lung cancer, respectively, and a total for the Danish population aged 50+.

A formal comparison of the estimated cost data for the different diseases requires an equal time horizon. Therefore, it is only relevant to compare the healthcare costs of cardiovascular diseases (CHD and stroke).

Stroke had a much higher potential of healthcare cost savings from air pollution mitigation compared to CHD. This is partly explained by the attributable healthcare costs per case, which are positive for any stroke age group and higher than those of CHD for any age/gender group, whereas it is negative for the oldest age groups of CHD. It is also partly due to the fact that the incidence of stroke is more responsive to changes in air pollution (i.e., has higher RRs) than is CHD.

### 3.1. Sensitivity Analysis

Tables 4–8 show sensitivity analyses of the total cost (corresponding to the last column of Table 3) when different model assumptions have been applied.

In the base case, we have used a modified Charlson index to control for comorbidity in the matching algorithm. In this first sensitivity analysis, we have excluded comorbidity from the matching algorithm in order to test the sensitivity of the base case results to comorbidity.  Table 4 shows the results of these calculations.

Table 4 shows us that potential healthcare cost savings increase marginally when excluding comorbidity from the matching. This indicates that comorbidity contributes in the propensity score of being incident with the four analysed diseases and by taking it out of the matching algorithm we select a set of controls that are healthier than the controls selected for the base case.

Both from a Danish [22] and a worldwide perspective [23], radical changes are expected to the composition of the population over the coming 50 years. All other things being equal, this will impact on potential healthcare cost savings.  Table 5 shows a one-way sensitivity analysis based on population projections from Statistics Denmark.

Estimates of the total healthcare cost savings were lower for the diseases, except for stroke, for which there was a close to zero change. The reduced healthcare cost savings is a result of a shift of the population towards a higher fraction of the older age groups and relatively smaller attributable healthcare costs in the older age groups.

It is a matter for discussion as to whether or not inclusion of savings to healthcare costs due to the premature death of case individuals is unethical. We have included these in the base case while some may argue that, by doing so, one favours disease and air pollution through potential healthcare cost savings due to increased mortality among cases. In order to shed light on the magnitude of this potential source of bias, we have calculated a one-way sensitivity analysis replacing ${\hat{S}}_{c}$ with ${\hat{S}}_{p}$ in the calculation of healthcare costs (6).  Table 6 shows the results of these calculations.

The results of the sensitivity analysis are that healthcare costs beyond the death of the cases are eliminated, giving a positive gain in the potential of reducing the levels of PM2.5. In absolute figures, there is a gain of €0.3–1.6 million in the potential healthcare cost savings. These are considerable changes in all four diseases, representing the fact that premature mortality is a major factor in these diseases (even when comorbidity is taken into account).

Table 7 shows a one-way sensitivity analysis of the healthcare costs of COPD associated with particulate emissions with updated survival on the prospective part. For survival in the year of the first hospitalisation, we used the 2006 cohort survival and adjusted the survival of the prospective part of the analysis on the basis of this.

The sensitivity analysis resulted in very small changes in the estimates of total healthcare cost savings compared to the base case. Updating the survival had little influence on the magnitude of total healthcare cost savings.

Our estimates of RRs taken from the literature were based on cohorts studied many years ago and under different circumstances; therefore, the RRs may not be representative for our case. Additionally, a mitigation of more than 50% of current air pollution levels may be difficult to achieve, especially considering that current levels are already below EU limit values [24]. It may be more realistic to consider scenarios with smaller changes in air pollution. Both these concerns relate to the RR and a sensitivity analysis of changes in RR therefore seemed evident.  Table 8 shows a scenario where mitigation is only 5 *μ*g/m^3^ (corresponding to half the RR).

In this scenario, healthcare costs of all four diseases decrease by 43% to 48%, indicating that the elasticity of the healthcare cost savings with respect to the RR is close to one in our model.

## 4. Discussion

We have devised a method to calculate the number of saved cases associated with a 10 *μ*g/m^3^ decline in the annual mean level of PM2.5 (using RRs found in the literature). We multiplied these numbers of cases with the average healthcare cost per episode of pollution related disease in order to achieve the healthcare cost savings attributable to a 10 *μ*g/m^3^ decline in the annual mean level of PM2.5. Using this method, it appears that there is a positive savings potential for the healthcare sector. However, the applied method is challenged by various factors.

The achievement of the objective to estimate potential benefits in terms of reduced healthcare costs rests on the assumption that all health effects of air pollution have been captured by the selection of the four diseases. This is not the case. More diseases than selected for this study were found to be related to air pollution [1]. However, the indirect method we used to identify exposed individuals requires that we only use diseases with significantly increased RRs of incidence or mortality, hence the selection of the four diseases. By not taking into account all potential benefits in terms of health effects, potential benefits in terms of healthcare costs represent an underestimation.

An important strength of the analysis is its basis in real-life individual data obtained from routine administrative registers. This enabled a much more detailed analysis and the combination of a number of very strong and well-proven methods and principles.

### 4.1. Applicability

The cost-benefit framework is useful for analysing the full effects of mitigating air pollution. In such a framework, the costs make up the actual costs of implementing mitigation interventions. The benefits would be in the form of healthcare cost savings, productivity gains and the value of increased utility of reduced pollution. The advantage of analysing in a cost-benefit framework is that it is intuitive and yields a single numeric (positive or negative) describing the net benefit of the mitigation effort. The results of our analysis may therefore be used as input in a cost-benefit framework. However, it is important to note that mitigating air pollution reduces incidence in diseases with high mortality, thereby increasing the average remaining life expectancy and hence the time span in which positive healthcare costs can be sustained. Combining this result with the productivity gains and the value of the increased utility of reduced pollution would give us the total benefits of an intervention, which reduces annual mean levels of PM2.5 by 10 *μ*g/m^3^. We have not considered the effects of mitigating domestic pollution on health abroad. Since air pollution is airborne, it may affect the health of neighbouring populations. Any such effects should also be incorporated in a cost-benefit analysis.

Our estimates were computed on the basis of Danish healthcare cost figures. They may therefore differ substantially from healthcare costs of pollution-related diseases in countries with different organisational healthcare sector and cost structures. We believe, however, that the method in itself is fully applicable to cost-benefit analyses in other countries.

### 4.2. Matching of Controls

We know that smoking, for instance, is a high-risk factor for the diseases analysed. However, smoking data are not included in the administrative registers. We explored the opportunity of matching with the inclusion of an index of smoking probability derived from a logistic regression from a subsample of the population. The controls identified by this approach had almost identical healthcare costs compared to controls identified without the smoking probability. Therefore, we abandoned this approach.

In the selection of controls for each disease, we excluded those individuals who became incident with the disease within the observation period. This may bias the estimates of healthcare cost for controls, since the control population may be “too healthy.” However, it was not possible to include them in the model using the method selected for this study. By including the individuals known to develop the disease, we get a control cohort that is “too ill.” A series of studies [25–27] discusses the issue of control selection.

In our matching, we found that CHD and stroke cases were slightly older than controls and lung cancer cases had higher comorbidity than controls. However, the absolute differences in age and comorbidity were relatively small and the significance is probably an indication that age did not contribute much to the propensity to be incident with CHD or stroke (already stratifying by five-year intervals) and that comorbidity did not contribute much to the propensity of being incident with lung cancer.

### 4.3. Relative Risks

The studies on which we based our assumptions of RRs (Table 3) might have been based on other levels of air pollution and in other populations. Application of these RRs for Denmark therefore relies heavily on the assumption that the RR is identical for other levels of air pollution. No evidence of a nonlinear relationship (within the ranges of PM pollution encountered in Europe) exists and for that reason a linear relationship is assumed.

It was not possible to establish the RRs of incidence of lung cancer associated with the outdoor concentration of PM2.5. Thus, we used the RRs of mortality as an approximation for those of incidence. This is not likely to induce much bias, since the prognosis of surviving once diagnosed with lung cancer is very poor.

### 4.4. Left Censoring, Left Truncation, and Time Horizon

We were challenged by left censoring in that some cases may have died prior to hospitalisation. However, by including information from the causes of death register, we believe that we captured the majority of cases. Cases particularly subject to left censoring are likely to be the mild cases that did not need hospitalisation and did not have the disease registered as cause of death. Most of the left censoring was likely to be in relation to COPD.

It was not possible to identify the date of onset of disease using register-based analysis. By using the first hospital contact (or time of death) to identify time of onset of disease, our analyses were subject to left truncation. Cases will always have had a period of time with the disease prior to the first hospital contact. We believed that the gravity of this error was largest for COPD, which is known to have a long period prior to hospitalisation. By using a nine-year prehospitalisation period for COPD cases, the error was assumed to be negligible. For the other three diseases, we believed that the error obtained from not including a pre-hospitalisation period was negligible.

Mortality was high for lung cancer. We therefore truncated the follow-up period of lung cancer to eight years to ensure sufficient statistical strength. Furthermore, we excluded individuals older than 79 years of age from the analysis of lung cancer. By truncating the follow-up period and excluding certain age groups, we disregarded an element of the costs in estimating the healthcare cost savings for lung cancer. Whether total healthcare cost savings would have been higher or lower depends on mortality and the level of healthcare costs of cases relative to controls in the years beyond the truncated period. This also applies to CHD, stroke, and COPD.

In accordance with the method used to define disease incidence and for reasons of limitations in data availability, our analyses were limited to 10, 10, 19, and eight years for CHD, stroke, COPD, and lung cancer, respectively. As previously noted, an extension of the time horizon may reduce or increase healthcare cost savings depending on mortality and healthcare costs of cases relative to controls.

### 4.5. Obsoletion of Data

Analysing healthcare costs of two cohorts of COPD patients and controls with different time of incidence informed us that survival and (initial) healthcare cost patterns had changed from 1997 to 2006. The 1997 cohort experienced lower survival rates and lower initial healthcare costs compared to the 2006 cohort. On the other hand, fewer patients were diagnosed in 2006 than in 1997. This same issue may apply to the other diseases if analysed prospectively.

## 5. Conclusion

To our knowledge, no previous study has used the combination of Lin's approach, attributable cost computation and register-based data to arrive at an expression of healthcare costs associated with a given exposure, in this case, air pollution.

In the present study, we succeeded in achieving our objective of developing a method for assessment of the benefits of mitigating air pollution in terms of healthcare cost savings, and applying it to the Danish case with fine particle matter. We found that there was a potential benefit in the form of reduced healthcare costs related to mitigation.

## Figures and Tables

**Table 1:** Characteristics of the populations at baseline.

	CHD	Stroke	COPD	COPD	Lung cancer
			before	after	
Number of cases	20,083	13,632	7,712	9,058	10,200
Age	72.1	73.9	71.3	70.2	67.0
Women (%)	46.4	52.7	51.9	50.3	42.4
Married (%)	55.3	48.6	48.9	55.1	61.1
Charlson score	0.3	0.3	0.4	0.3	1.2
Education (%)					
Unskilled	74.9	78.6	63.2	76.6	64.8
Skilled	18.2	15.5	28.0	18.3	26.8
Higher education	6.9	5.9	8.8	5.2	8.4
Socioeconomic status (%)					
White collar	6.7	4.6	2.9	3.6	5.2
Blue collar	10.5	7.3	9.7	7.4	11.3
Unemployed	1.7	1.3	1.6	1.8	1.7
Outside labour market	81.1	86.9	85.8	87.2	81.8

**Table 2:** Incidence per 100,000 inhabitants and attributable healthcare costs per case (€, 2006 level).

	50–54	55–59	60–64	65–69	70–74	75–79	80+
Incidence per 100,000 inhabitants							
CHD							
Men	409	610	850	1,113	1,478	1,936	3,125
Women	131	242	350	551	764	1,224	2,746
Stroke							
Men	240	388	620	867	1,236	1,746	2,473
Women	144	221	348	590	854	1,372	2,286
COPD							
Men	155	198	318	511	834	944	851
Women	169	217	335	481	680	787	523
Lung cancer							
Men	88	151	283	409	524	517	
Women	73	114	206	275	274	252	

Attributable healthcare cost per case							
CHD							
Men	21,650	21,466	20,247	12,249	6,080	−2,062	−9,991
Women	19,365	21,177	18,711	14,907	10,398	−489	−10,360
Stroke							
Men	32,924	33,178	31,586	27,785	19,640	15,191	3,994
Women	30,212	30,850	32,534	25,847	22,975	13,795	1,512
COPD							
Men	23,315	27,786	32,130	28,859	22,177	9,869	4,427
Women	31,174	32,698	41,222	36,695	26,390	18,834	11,636
Lung cancer							
Men	33,515	23,685	22,467	9,207	4,111	−2,905	
Women	33,371	24,508	17,831	13,485	4,590	−3,522	

**Table 3:** Healthcare cost savings attributable to a 10 *μ*g/m^3^ decline in annual mean PM2.5 (million €, 2006 level).

	RR	50–54	55–59	60–64	65–69	70–74	75–79	80+	Total*
CHD									
Men	1.105	0.8	1.2	1.6	1.3	0.9	−0.4	−3.0	0.7
Women	1.21	0.4	0.9	1.1	1.4	1.4	−0.1	−4.4	0.1
Stroke									
Men	1.175	1.2	1.9	2.9	3.6	3.6	3.9	1.5	2.5
Women	1.35	1.1	1.8	2.9	4.0	5.1	4.9	0.9	2.6
COPD									
Men	1.14	0.4	0.7	1.3	1.8	2.3	1.1	0.5	1.1
Women	1.14	0.6	0.9	1.7	2.2	2.2	1.8	0.7	1.3
Lung cancer									
Men	1.14	0.4	0.4	0.8	0.5	0.3	−0.2		0.4
Women	1.14	0.3	0.3	0.5	0.5	0.2	−0.1		0.3

Note: the denominator of healthcare costs per age group is based on the Danish age composition at baseline.

*Total is a weighted total for all age groups.

**Table 4:** Sensitivity analysis based on matching without co-morbidity (million €, 2006 level).

	RR	Total	Deviation	(%)
CHD				
Men	1.105	0.9	0.2	(25.9)
Women	1.21	0.3	0.2	(218.9)
Stroke				
Men	1.175	2.8	0.3	(12.8)
Women	1.35	3.0	0.3	(12.9)
COPD				
Men	1.14	1.3	0.2	(21.8)
Women	1.14	1.5	0.2	(14.5)
Lung cancer				
Men	1.14	0.6	0.2	(41.7)
Women	1.14	0.4	0.1	(42.1)

**Table 5:** Sensitivity analysis—2050 population standards (million €, 2006 level).

	RR	Total	Deviation	(%)
CHD				
Men	1.105	0.1	−0.6	(−85.7)
Women	1.21	−0.4	−0.5	(−497.9)
Stroke				
Men	1.175	2.5	0.0	(0.5)
Women	1.35	2.6	0.0	(0.5)
COPD				
Men	1.14	1.1	0.0	(−1.7)
Women	1.14	1.3	0.0	(−0.7)
Lung cancer				
Men	1.14	0.4	−0.1	(−15.6)
Women	1.14	0.3	0.0	(−11.6)

**Table 6:** Sensitivity analysis based on case survival (million €, 2006 level).

	RR	Total	Deviation	(%)
CHD				
Men	1.105	1.4	0.7	(99.7)
Women	1.21	1.3	1.1	(1096.8)
Stroke				
Men	1.175	3.3	0.8	(33.6)
Women	1.35	4.2	1.6	(61.3)
COPD				
Men	1.14	1.5	0.5	(42.2)
Women	1.14	1.7	0.4	(26.8)
Lung cancer				
Men	1.14	0.9	0.5	(113.0)
Women	1.14	0.6	0.3	(96.3)

**Table 7:** Sensitivity analysis—COPD survival (million €, 2006 level).

	RR	Total	Deviation	(%)
COPD				
Men	1.14	1.1	0.0	(2.3)
Women	1.14	1.4	0.0	(2.5)

**Table 8:** Sensitivity analysis: 5 *μ*g/m^3^ decrease in the annual mean level of PM2.5 (million €, 2006 level).

	RR	Total	Deviation	(%)
CHD				
Men	1.088	0.4	−0.3	(−47.5)
Women	1.105	0.1	0.0	(−45.2)
Stroke				
Men	1.053	1.3	−1.1	(−46.0)
Women	1.175	1.5	−1.1	(−42.6)
COPD				
Men	1.07	0.6	−0.5	(−46.7)
Women	1.07	0.7	−0.6	(−46.7)
Lung cancer				
Men	1.07	0.2	−0.2	(−46.7)
Women	1.07	0.2	−0.1	(−46.7)
